# RNF31 promotes proliferation and invasion of hepatocellular carcinoma via nuclear factor kappaB activation

**DOI:** 10.1038/s41598-023-50594-3

**Published:** 2024-01-03

**Authors:** Kouki Hoshino, Seshiru Nakazawa, Takehiko Yokobori, Kei Hagiwara, Norihiro Ishii, Mariko Tsukagoshi, Takamichi Igarashi, Kenichiro Araki, Norifumi Harimoto, Fuminori Tokunaga, Ken Shirabe

**Affiliations:** 1 Department of General Surgical Science, Gunma University Graduate School of Medicine, 3-39-22 Showa-machi, Maebashi, 371-8511 Japan; 2 Gunma University Initiative for Advanced Research, Maebashi, Japan; 3 Department of Medical Biochemistry, Graduate School of Medicine, Osaka Metropolitan University, Osaka, Japan

**Keywords:** Hepatocellular carcinoma, Hepatocellular carcinoma

## Abstract

RNF31 is a multifunctional RING finger protein implicated in various inflammatory diseases and cancers. It functions as a core component of the linear ubiquitin chain assembly complex (LUBAC), which activates the nuclear factor kappaB (NF-κB) pathway via the generation of the Met1-linked linear ubiquitin chain. We aimed to clarify the role of RNF31 in the pathogenesis of hepatocellular carcinoma (HCC) and its relevance as a therapeutic target. High RNF31 expression in HCC, assessed by both immunohistochemistry and mRNA levels, was related to worse survival rates among patients with HCC. In vitro experiments showed that RNF31 knockdown in HCC cell lines led to decreased cell proliferation and invasion, as well as suppression of tumor necrosis factor (TNF)-α-induced NF-κB activation. Treatment with HOIPIN-8, a specific LUBAC inhibitor that suppresses RNF31 ubiquitin ligase (E3) activity, showed similar effects, resulting in decreased cell proliferation and invasion. Our clinical and in vitro data showed that RNF31 is a prognostic factor for HCC that promotes tumor aggressiveness via NF-κB activation.

## Introduction

Hepatocellular carcinoma (HCC) is the sixth most common cancer in the world and is the fourth leading cause of cancer-related deaths^1–3^. Treatment strategies for HCC have significantly evolved with advances in diagnostic modalities, surgical techniques, and systemic therapies^1,2,4^. However, the incidence of HCC continues to increase, and a complete cure remains challenging^1,2,5,6^. Therefore, to improve the prognosis of HCC patients, better biomarkers and new therapeutic targets are needed.

A major factor contributing to the pathogenesis of HCC is cirrhosis, which results from chronic inflammation caused by hepatitis or fatty liver^7,8^. Inflammation itself is also associated with a poor prognosis for patients with HCC^9^. The regulation of inflammation by the nuclear factor kappaB (NF-κB) pathway has been shown to promote the proliferation of many cancers, including HCC^10–13^. Amongst various regulators of the NF-κB pathway, the linear ubiquitin chain assembly complex (LUBAC) has been identified as a unique enzymatic complex that regulates the NF-κB pathway by linear ubiquitination of downstream effectors^14,15^. RING finger protein 31 (RNF31, also known as HOIP) functions as the catalytic center of LUBAC and forms a complex with RBCK1 (RANBP2‐type and C3HC4‐type zinc finger containing 1 or HOIL-1L) and SHANK-associated RH domain-interacting protein (SHARPIN) subunits. Importantly, RNF31 can also function as a single molecule by interacting with estrogen receptor α (ERα) or via the p53 pathway^16–20^. The role of RNF31 in the context of NF-κB activation in HCC remains unclear. In this study, we aimed to evaluate the prognostic value of RNF31 and investigate its potential as a therapeutic target for HCC.

## Results

### RNF31 expression is associated with poor survival in patients with HCC

**Figure 1:**
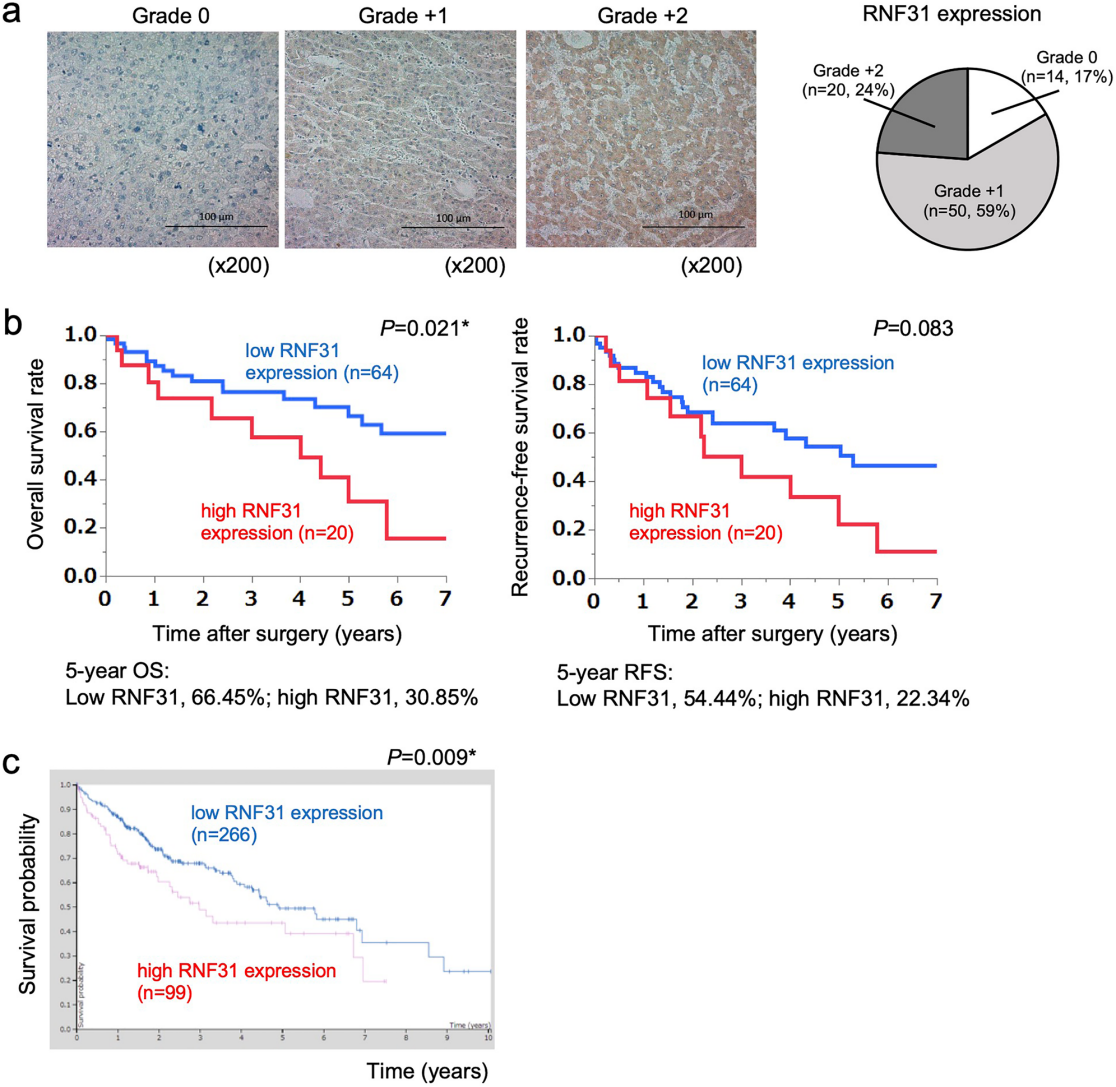
Correlation between RNF31 expression and survival of patients with hepatocellular carcinoma. (**a**) Representative immunohistochemical (IHC) staining of RNF31 in cancerous areas of an HCC specimen (original magnification, ×200). RNF31 expression was scored as grades 0, +1, and +2 according to staining intensity. (**b**) Overall survival and recurrence-free survival according to RNF31 IHC expression. (**c**) Survival according to RNF31 mRNA expression in The Cancer Genome Atlas database of patients with liver cancer (image credit: Human Protein Atlas, image available from v22.0 proteinatlas.org). OS, overall survival; RFS, recurrence-free survival.

First, we analyzed the relationship between RNF31 expression and the survival of patients with HCC who underwent surgical resection at our institute (n = 84). The localization of RNF31 protein was cytoplasmic based on immunohistochemical (IHC) staining. The number of cases classified in RNF31 expression grades 0/+1/+2 were 14/50/20, respectively (Fig. 1a). Sixty-four patients were classified as the low RNF31 expression group (grades 0 and +1) and 20 patients were classified in the high RNF31 expression group (grade +2). The 5-year overall survival rate was significantly better in the low RNF31 expression group than in the high RNF31 expression group (66.45% vs. 30.85%; *P* = 0.021) (Fig. 1b). The low RNF31 expression group had a better 5-year recurrence-free survival rate than the high RNF31 expression group (54.44% vs. 22.34%; *P* = 0.083). There was also a significant difference in overall survival when RNF31 expression was stratified into grades 0, +1, and +2 groups (*P* = 0.028) (Fig. S1). We confirmed these findings by analyzing the relationship between RNF31 mRNA expression and survival using data from The Cancer Genome Atlas database (n = 365) (Fig. 1c)^21^. Consistent with IHC results, high RNF31 mRNA expression was associated with significantly worse survival (*P* = 0.009).

### RNF31 is a negative prognostic factor for HCC

We analyzed the relationships between clinicopathological factors and RNF31 expression (Table 1). Only the Ki-67 index showed a significant difference between the high and low RNF31 expression groups (*P* = 0.040). We found no correlation between RNF31 expression and viral hepatitis or liver cirrhosis (*P* = 0.824 and *P* = 0.653, respectively). Our univariate analysis indicated that high RNF31 expression was a prognostic factor associated with poor survival (hazard ratio [HR], 2.31; 95% confidence interval [CI] 1.07–5.00; *P* = 0.034), as well as tumor classification and tumor growth patterns (*P* = 0.001 and *P* = 0.002, respectively) (Table 2). These three prognostic factors were also independent risk factors according to our multivariate analysis.

**Table 1:** Clinicopathological characteristics of hepatocellular carcinoma patients according to RNF31 expression.

Patient characteristics	RNF31		*P* value
	Low expression (n = 64)	High expression (n = 20)	
Age (y)			
≤ 65	21	3	0.124
> 65	43	17	
Sex			0.351
Male	48	17	
Female	16	3	
Viral hepatitis			0.824
Negative	27	9	
Positive	37	11	
Liver cirrhosis			0.653
Negative	38	13	
Positive	26	7	
Differentiation			0.837
Well or moderate	60	19	
Poor	4	1	
T classification			0.154
1	6	2	
2	16	9	
3	32	9	
4	10	0	
Tumor growth pattern			0.544
Expansive growth	52	15	
Invasive growth	12	5	
Cancerous capsule infiltration			0.065
Negative	33	15	
Positive	31	5	
Intrahepatic metastasis			0.109
Negative	51	19	
Positive	13	1	
Portal vein invasion			0.118
Negative	39	16	
Positive	25	4	
Hepatic vein invasion			0.901
Negative	52	16	
Positive	12	4	
Hepatic artery invasion			0.252
Negative	60	20	
Positive	4	0	
Tumor size (mm)			0.478
≤ 20	6	3	
> 20	58	17	
AFP (ng/mL)	992 ± 5089	239 ± 1015	0.286
PIVKA-II (mAU/mL)	4464 ± 17,558	18,686 ± 71,201	0.388
CD8 infiltration			0.466
Low (≤ 10)	17	7	
High (> 10)	47	13	
Ki-67 labelling index			0.040*
≤ 10	57	14	
> 10	7	6	

*AFP* α-fetoprotein; *PIVKA-II* protein induced by vitamin K absence II.

*P < 0.05.

**Table 2:** Univariate and multivariate analyses of RNF31 expression and clinicopathological features of patients with hepatocellular carcinoma.

Clinicopathologic variables	Univariate analysis			Multivariate analysis		
	HR	95% CI	*P* value	HR	95% CI	*P* value
Age (y) (≤ 65/ > 65)	1.17	0.52–2.66	0.701	–	–	–
Sex (male/female)	1.13	0.48–2.62	0.780	–	–	–
Virus (nonvirus/virus)	0.85	0.41–1.74	0.659	–	–	–
Liver cirrhosis (negative/positive)	1.86	0.91–3.77	0.087	–	–	–
Differentiation (well or moderate/poor)	1.30	0.31–5.49	0.723	–	–	–
Tumor classification (T1–T3, T4)	4.33	1.82–10.34	0.001*	5.92	2.24–15.64	< 0.001*
Tumor growth pattern (expansive/invasive)	3.29	1.53–7.07	0.002*	2.45	1.10–5.48	0.029*
Tumor size (≤ 20 mm/ > 20 mm)	1.18	0.41–3.40	0.757	–	–	–
Ki-67 labelling index (≤ 10/ > 10)	1.46	0.63–3.39	0.379	–	–	–
RNF31 expression (low/high)	2.31	1.07–5.00	0.034*	2.69	1.11–6.54	0.029*

*HR* hazard ratio; *CI* confidence interval; *AFP* α-fetoprotein; *PIVKA-II* protein induced by vitamin K absence II.

*P < 0.05.

### Relation between RNF31 expression in cancerous lesion and background liver tissue

Since RNF31 expression in underlying liver tissue may impact HCC pathogenesis, we analyzed RNF31 expression in the background liver tissue as well. We first compared the relationship between RNF31 expression in cancerous lesions and background liver tissue (Fig. S2a). RNF31 expression was significantly higher in cancerous lesions than in background liver tissue (*P* < 0.05, Wilcoxon signed-rank test). Most of the cases had low RNF31 expression in both background liver and HCC (53/82, 64.6% of total cases), whereas only one case had high RNF31 expression in both background liver and HCC (1/82, 1.2%). For the remaining 28 cases (34.1%), RNF31 expression was high in HCC but low in background liver tissue, or low in HCC but high in background liver tissue. Fig. S2b shows a representative example of a paired sample from the same patient with high RNF31 expression in the cancer lesion and low RNF31 expression in the background liver tissue. We also analyzed clinicopathological factors according to RNF31 expression in the background liver tissue (Supplementary Table S1). In patients with high RNF31 expression in the background liver tissue, invasive growth pattern, portal vein invasion, and hepatic artery invasion occurred significantly more often (*P* = 0.03, *P* = 0.005, and *P* = 0.028, respectively). There was no correlation between RNF31 expression in background liver tissue and the presence of viral hepatitis or liver cirrhosis (*P* = 0.588 and *P* = 0.574, respectively). To clarify the expression of RNF31 in normal liver, we identified within our cohort HCC cases that had no clinically relevant underlying disease in the background liver, such as cirrhosis, chronic inflammation, fibrosis, or viral infection. There were nine cases with normal background liver, comprising three cases (33.3%, 3/9) with high RNF31 expression in the background liver tissue and six cases (66.7%, 6/9) with low RNF31 expression.

### RNF31 knockdown suppresses the proliferation and invasion of HCC cell lines.

**Figure 2:**
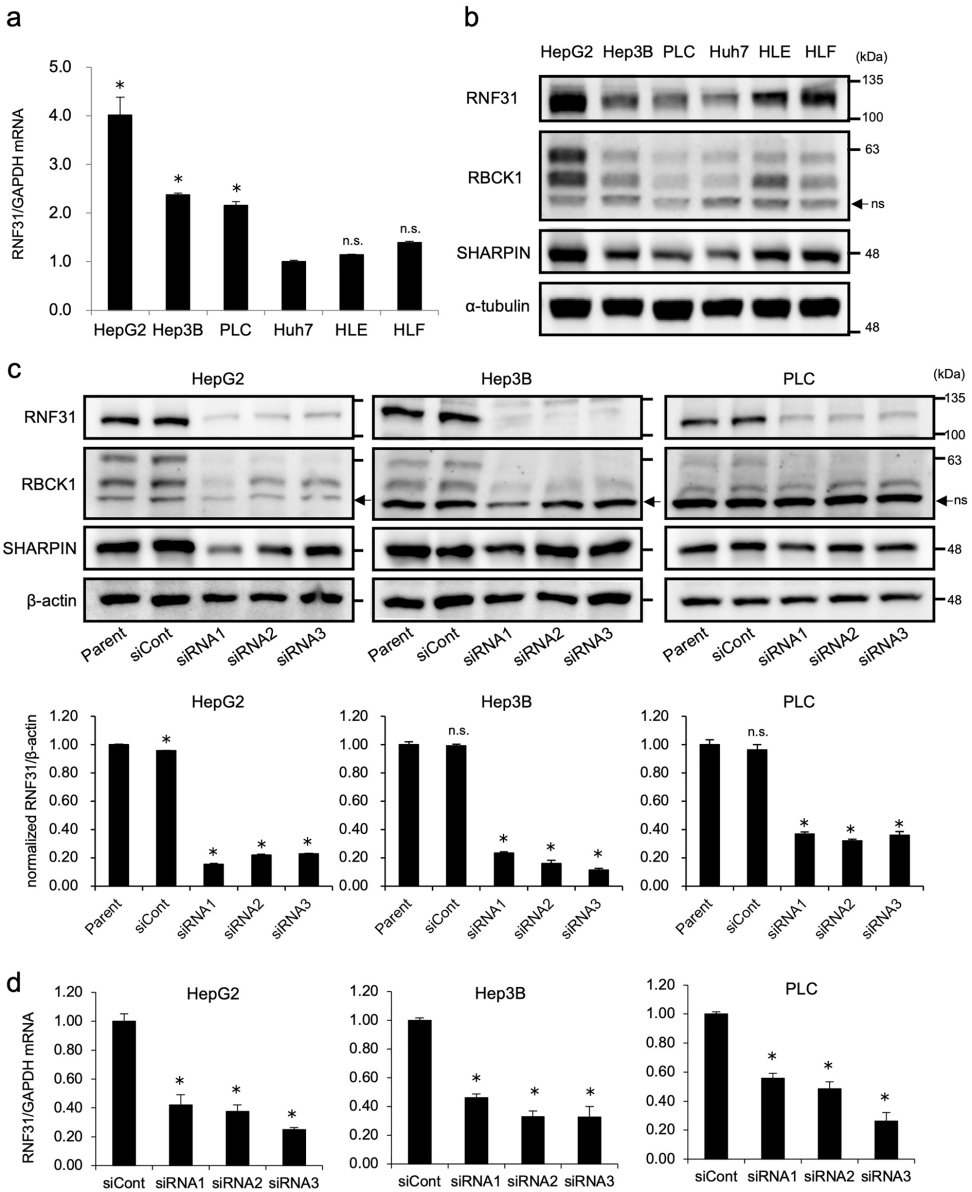
Expression of RNF31 in HCC cell lines and evaluation of RNF31 knockdown. (**a**) The mRNA level of RNF31 was evaluated in the HepG2, Hep3B, PLC, Huh7, HLE, and HLF cell lines by qPCR. mRNA level of RNF31 in Huh7 cells was used as reference. (**b**) The protein expression levels of RNF31, RBCK1, and SHARPIN were evaluated by Western blotting. (**c**) The effect of RNF31 knockdown was evaluated using Western blotting for HepG2, Hep3B, and PLC cells transfected with control (siCont) and RNF31-specific siRNAs (siRNA1-3). For quantification, normalized RNF31/b-actin level in parental cells was used as reference. d. The effect of RNF31 knockdown was evaluated using qPCRs. mRNA level of RNF31/GAPDH in control cells was used as reference. *P < 0.05; ns, non-specific; n.s., not significant; siCont, control siRNA.

**Figure 3:**
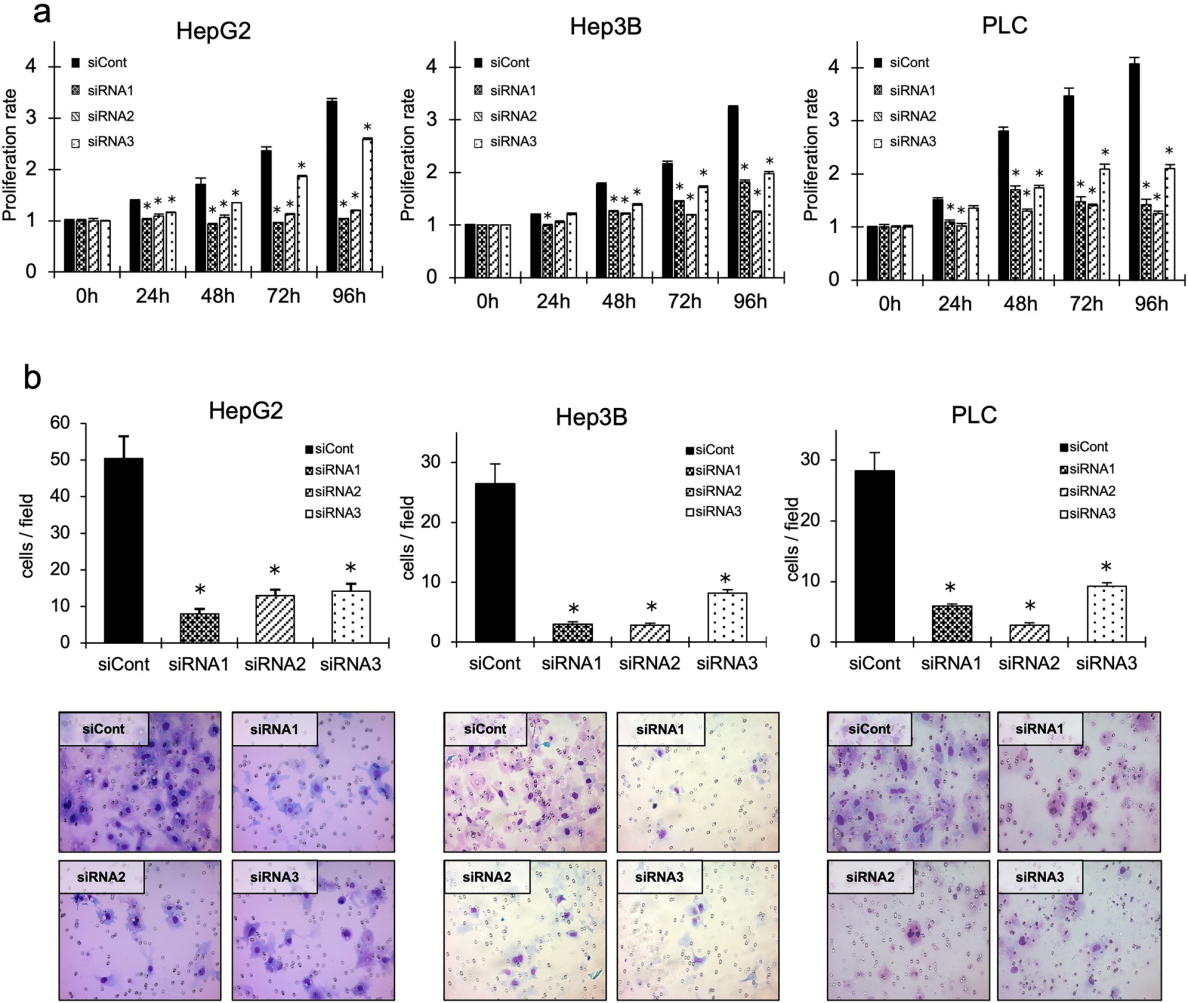
Knockdown of RNF31 leads to decreased cell proliferation and invasion in HCC cell lines. (**a**) The effect of RNF31 knockdown on the proliferation rate of HepG2, Hep3B, and PLC cells was evaluated at 24, 48, 72, and 96 h after siRNA transfection. (**b**) The effect of RNF31 knockdown on invasion of HepG2, Hep3B, and PLC cells was evaluated at 48 h after seeding cells in the invasion chambers. Representative images of invading cells are shown in the lower panel. Cell nuclei are stained in purple. *P < 0.05; siCont, control siRNA.

The in vitro effects of RNF31 on HCC cells were analyzed next. First, we evaluated the mRNA and protein levels of RNF31 in six HCC cell lines, in addition to RBCK1 and SHARPIN, the two other components of LUBAC. RNF31 mRNA expression was highest in HepG2 cells, followed by Hep3B and PLC cells (Fig. 2a). Protein expression of RNF31 was also highest in HepG2 cells, followed by HLF and Hep3B cells (Fig. 2b, Fig. S3a). Both mRNA and protein expression of RBCK1 were highest in HepG2 cells (Fig. S4a, Fig. 2b, Fig. S3b). SHARPIN mRNA expression levels were particularly high in Hep3B and PLC, followed by HepG2 cells (Fig. S4b). SHARPIN protein expression was highest in HepG2 cells (Fig. 2b, Fig. S3c). For subsequent experiments, we selected HepG2, Hep3B, and PLC cells, in which the mRNA level of RNF31 was high and the other LUBAC components, RBCK1 and SHARPIN, were expressed in a balanced pattern. We performed RNF31 knockdown using three independent siRNAs, which induced a substantial reduction of RNF31 mRNA and protein expression in HepG2, Hep3B, PLC (Fig. 2c,d). RNF31 knockdown also reduced the protein level of RBCK1 in HepG2, Hep3B, and PLC (Fig. S5a). Protein level of SHARPIN was also decreased by RNF31 knockdown in HepG2 and PLC, but not in Hep3B cells (Fig. S5b). In all three cell lines, RNF31 knockdown significantly decreased the cell proliferation rate (*P* < 0.05) (Fig. 3a). Invasion was also significantly suppressed in RNF31-silenced cells (*P* < 0.05) (Fig. 3,3,3,3b).

### RNF31 enhances NF-κB activation in HCC cell lines.

**Figure 4:**
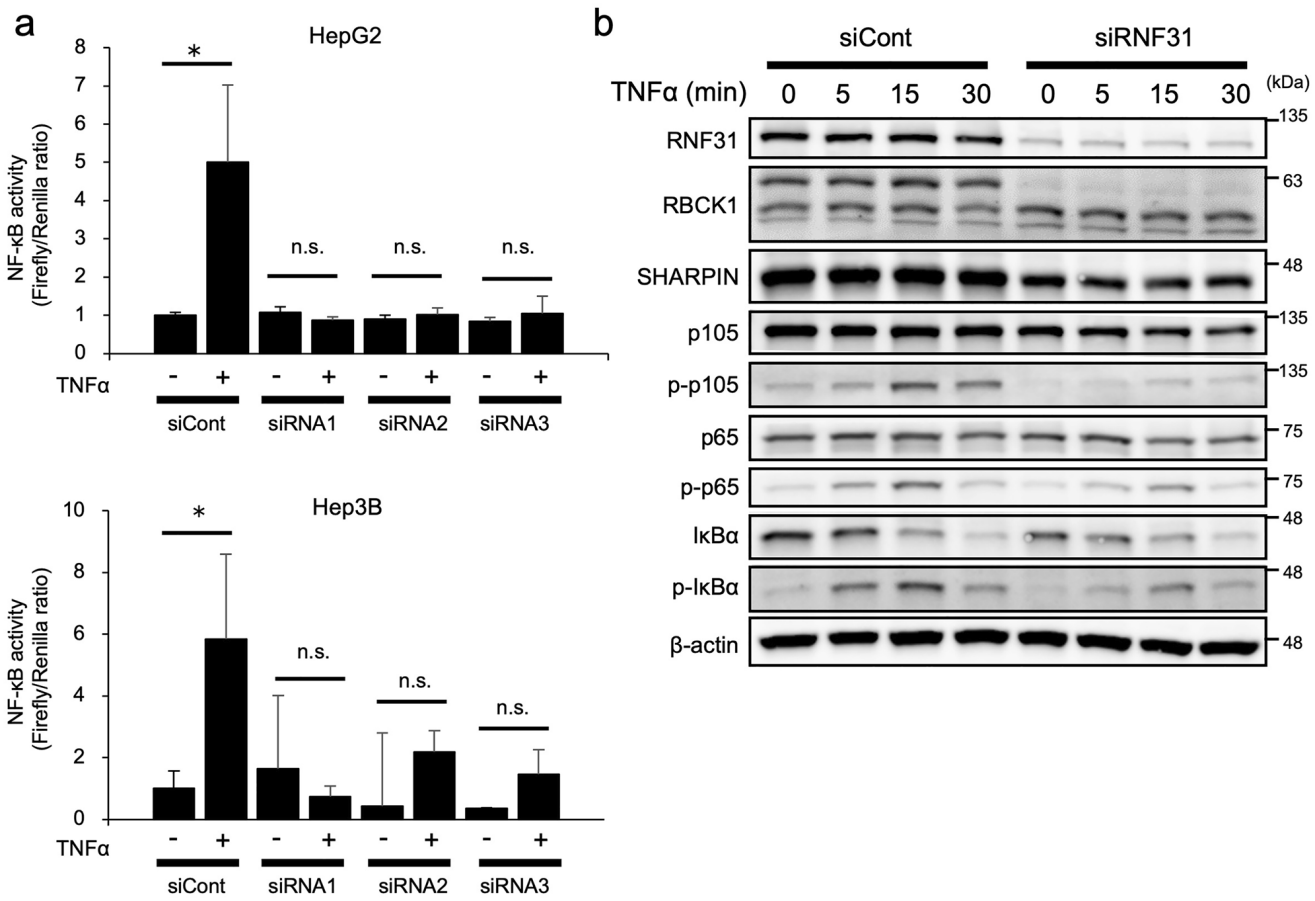
RNF31 knockdown inhibits TNF-α-induced NF-κB activation in HCC cell lines. (**a**) NF-κB activity was evaluated in HepG2 and Hep3B cells transfected with RNF31 siRNAs using a dual luciferase assay. Cells were treated with TNF-α at 10 ng/mL for 6 h. (**b**) Evaluation of phosphorylation of NF-κB signaling factors induced by TNF-α in HepG2 cells after RNF31 knockdown. *P < 0.05; n.s., not significant; siCont, control siRNA.

We assessed the effect of RNF31 knockdown on the NF-κB pathway. We first examined the activation of NF-κB by tumor necrosis factor (TNF)-α using a dual luciferase assay in HepG2 and Hep3B cells. As expected, TNF-α stimulation induced NF-κB activation of control cells, whereas RNF31 knockdown significantly suppressed NF-κB activation (Fig. 4a). Accordingly, upon TNF-α stimulation, phosphorylation of the NF-κB signaling factors, such as p105, p65, and IκBα, was also attenuated in RNF31-silenced HepG2 cells (Fig. 4b, Fig. S6), indicating that RNF31 knockdown inhibited TNF-α-induced activation of NF-κB signaling pathway in HCC cell lines.

### HOIPIN-8 suppresses the proliferation and invasive potential of HCC cell lines.

**Figure 5:**
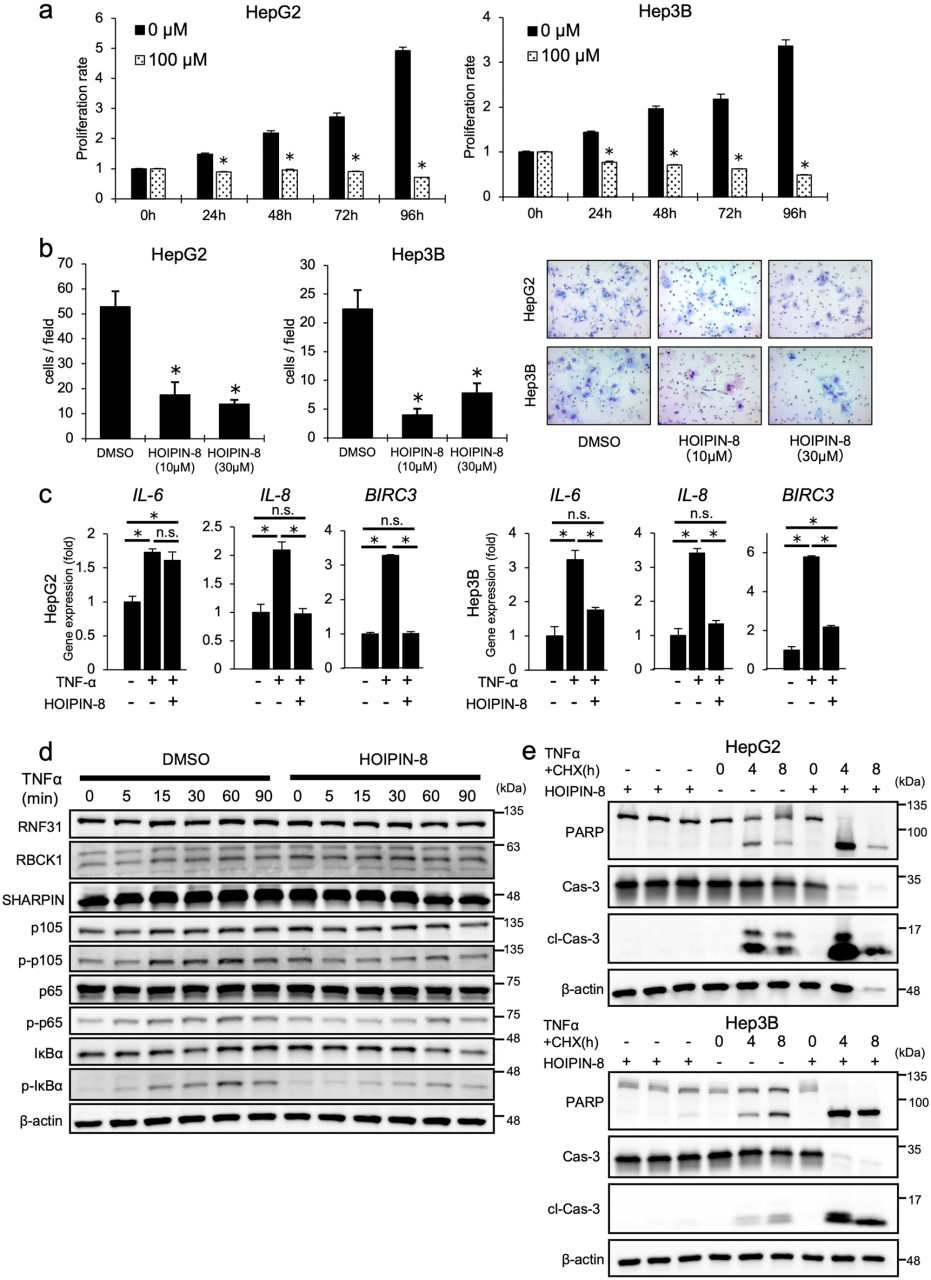
Inhibition of RNF31 with HOIPIN-8 decreases proliferation, invasion, and NF-κB activation in HCC cell lines. (**a**) Cell proliferation was evaluated in HepG2 and Hep3B cells treated with 100 µM HOIPIN-8. (**b**) Invasion was evaluated in HepG2 and Hep3B cells in the presence of 10 or 30 µM HOIPIN-8. Representative images of invading cells are shown in the right panel. Cell nuclei are stained in purple. (**c**) Expression of NF-κB target genes in HepG2 and Hep3B cells was evaluated by qPCR. Cells were pretreated with 30 µM HOIPIN-8 for 1 h, followed by stimulation with 10 ng/mL TNF-α for 2 h. (**d**) Phosphorylation of NF-κB signaling factors upon TNF-α stimulation was evaluated in HepG2 cells with or without 30 µM HOIPIN-8. Cell lysates were immunoblotted by the indicated antibodies. (**e**) Effect of HOIPIN-8 on TNF-α + CHX-mediated apoptosis in HepG2 and Hep3B cells. Cells were pre-treated with HOIPIN-8 for 1 h and then stimulated with 40 ng/mL TNF-α and 20 µg/mL CHX for 0, 4, and 8 h, and cell lysates were immunoblotted by the indicated antibodies. *P < 0.05; n.s., not significant; Cas-3, caspase-3; cl-Cas-3, cleaved caspase-3; CHX, cycloheximide.

Next, we assessed the effects of HOIPIN-8, an α,β-unsaturated carbonyl-containing LUBAC-specific inhibitor^22,23^, on HCC cell lines. Similar to RNF31 knockdown, HOIPIN-8 significantly suppressed the proliferation rate of HCC cell lines (*P* < 0.05) (Fig. 5a). The IC50 of HOIPIN-8 on HepG2 and Hep3B cells was about 70–100 µM (Supplementary Table S2) and invasion was significantly suppressed at lower concentrations such as 10 or 30 µM. (*P* < 0.05) (Fig. 5b). HOIPIN-8 further suppressed the induction of NF-κB target genes (*interleukin [IL]-6*, *IL-8*, and *BIRC3*) (Fig. 5c) and the phosphorylation of NF-κB signaling factors upon TNF-α stimulation (Fig. 5d, Fig. S7). To understand if the effect of HOIPIN-8 on HCC cell lines was reversible, HOIPIN-8 was washed out after 24 h, and the subsequent proliferation rate was analyzed for 96 h. Cells that had wash-out of HOIPIN-8 showed a small but significantly higher proliferation rate when compared to cells which underwent continuous treatment with HOIPIN-8 (*P* < 0.05, Fig. S8). Lastly, we verified whether HOIPIN-8 could affect apoptosis induced by TNF-α and cycloheximide. The combined treatment of HOIPIN-8 with TNF-α and cycloheximide markedly enhanced the cleavage of PARP and caspase 3, suggesting that induction of the extrinsic apoptotic pathway could be enhanced by HOIPIN-8 (Fig. 5e). Similarly, the rate of apoptosis using the Annexin V/PI apoptosis assay showed that late apoptosis was significantly increased in the combination treatment of HOIPIN-8 with TNF-α and cycloheximide (Fig. S9).

## Discussion

The key findings of our study were as follows: RNF31 was associated with poor survival and was an independent prognostic factor for HCC. Knockdown of RNF31 decreased proliferation and invasion in HCC cell lines with decreased NF-κB activation upon TNF-α stimulation. Treatment with HOIPIN-8, a LUBAC-specific inhibitor, had similar inhibitory effects on cell proliferation, invasion, and NF-κB activation as those of RNF31 knockdown. Based on these results, we concluded that RNF31 is a prognostic biomarker and a potential therapeutic target for HCC.

Our analysis of patients with HCC showed a significant correlation between survival and RNF31 expression as assessed by immunohistochemistry. These findings were also confirmed by an analysis of The Cancer Genome Atlas database, which uses mRNA levels to evaluate RNF31 expression. Because RNF31 plays an important role in inflammatory signaling as a component of LUBAC, we initially expected that RNF31 could be related to an inflammatory background, such as liver cirrhosis or chronic viral infections. Shimizu et al. reported that depletion of RNF31 led to chronic inflammation and apoptosis in mouse liver parenchyma, resulting in increased hepatocarcinogenesis^24^. However, in our cohort, RNF31 expression was not correlated with viral hepatitis or liver cirrhosis. Chen et al. also reported that there was no correlation between RNF31 expression and HBV or cirrhosis^25^.

As a mechanism of RNF31 in HCC, Chen et al. suggested that RBCK1 could promote HCC metastasis and growth by stabilizing RNF31^25^. Because RNF31 and RBCK1 are both essential components of the LUBAC, we suspected that the stabilization of RNF31 by RBCK1 could increase LUBAC formation and subsequently trigger enhanced NF-κB activation via linear ubiquitination. Consistent with this, Chen et al. showed that gliotoxin, an inhibitor of LUBAC activity, has inhibitory effects on HCC cell lines^25^. However, they did not clarify whether the inhibitory effect of gliotoxin was due to LUBAC or NF-κB activity, as gliotoxin itself has cytotoxicity^23^. In this study, we showed that RNF31 knockdown could actually inhibit TNF-α-induced NF-κB activation. Furthermore, we used HOIPIN-8, a LUBAC-specific inhibitor, which suppresses the E3 activity of RNF31 by modifying the active Cys885^22,23^. This leads to robust inhibition of both LUBAC activation and the NF-κB pathway with low cytotoxicity^23^. Similar to the results of RNF31 knockdown, the treatment of HCC cell lines with HOIPIN-8 also resulted in decreased cell proliferation, invasion, NF-κB activation, and enhanced apoptosis. Therefore, reducing the expression and activity of LUBAC can be considered as effective treatment for HCC.

NF-κB plays a crucial role in cancer development and progression^10,13,26^. Therefore, various NF-κB inhibitors have been developed and investigated for their anti-tumor effects. However, the clinical application of these inhibitors is limited due to side effects and issues related to drug dosage. In addition to their effect on tumors, NF-κB inhibitors may reduce systemic immunity to infections and other diseases and, more importantly, could negatively impact anti-tumor immunity. In this study, we used HOIPIN-8, a refined derivative of HOIPIN-1, which was developed as a specific inhibitor of LUBAC. HOIPIN-1 and -8 effectively induced cell death in activated B cell-like diffuse large B cell lymphoma cells (ABC-DLBCL)^22,23^. In addition to its anti-tumor effect, HOIPIN-1 could enhance anti-tumor immunity in melanoma models, even when T cells were pretreated with HOIPIN-1^27^. Interestingly, Frey et al. showed that RNF31 depletion in pancreatic ductal carcinoma cell lines could increase both the infiltration and effector functions of CD8^+^ T cells in an orthotopic tumor model^28^. Unfortunately, we could not validate this effect of RNF31 on anti-tumor immunity in our cohort of patients with HCC, since we identified no correlation between tumor-infiltrating CD8^+^ cells and levels of RNF31 expression (Table 1).

In conclusion, our study demonstrated that RNF31 expression is an important prognostic factor for HCC, and RNF31 is involved in HCC tumor progression via the NF-κB pathway. These results suggest that RNF31 may be a potential therapeutic target for HCC as well as a novel biomarker. One limitation of this study was that it was a single-center, retrospective study with a limited sample size, which may have biased the clinical results. To validate the therapeutic potential of RNF31 against HCC, further large-scale studies are needed. Additionally, in vivo studies are required to clarify the interactions between RNF31 and the immune environment.

## Materials and methods

### Patients and samples

We analyzed tumor tissues of 84 patients with HCC who underwent surgical treatment at the Department of General Surgical Science Gunma University (Maebashi, Japan) between 1996 and 2014. The tumor stage was classified according to the 6th Japanese Tumour-Node-Metastasis (TNM) Classification of the Liver Cancer Study Group of Japan^29^. All clinical samples and patient data were analyzed following Gunma University Hospital’s institutional guidelines (approval number: HS2020-124) and the Declaration of Helsinki, with a waivor of informed consent, using the opt-out method. All experimental protocols were approved by Gunma University Ethical Review Board for Medical Research Involving Human Subjects. Patient consent was obtained by using the opt-out method.

### Tissue microarrays

Formalin-fixed paraffin-embedded samples obtained from HCC patients were stored in the Clinical Department of Pathology at Gunma University Hospital. The formalin-fixed paraffin-embedded tissue blocks were marked with two representative tumor areas after examining the slides stained with hematoxylin and eosin. Tumor cores with a diameter of 2.0 mm were removed using a cylinder. A manual arraying instrument (Beecher Instruments, Sun Prairie, WI, USA) was used to assemble paraffin blocks into tissue microarrays as previously described^30^.

### Immunohistochemistry analysis

The tissue microarray blocks were cut into 4-µm-thick slices, and immunostaining was performed using a primary antibody against RNF31 (1:200; anti-HOIP antibody ab133818; Abcam, Cambridge, UK). Each mounted section was de-paraffinized, rehydrated, and incubated with 0.3% hydrogen peroxide in methanol for 30 min at room temperature to block the endogenous peroxidase activity. Antigen retrieval for RNF31 was performed by boiling slides in 10 mmol/L citrate buffer (pH 6.0) at 98 ℃ for 45 min. Non-specific binding sites were blocked for 30 min at room temperature using Protein Block Serum-Free (Dako, Osaka, Japan). The sections were then incubated with a primary antibody for 24 h at 4 ℃. Following washing with phosphate-buffered saline (PBS), they were coated with Histofine Simple Stain MAX‐PO (Multi) Kit (Nichirei Biosciences, Tokyo, Japan) for 1 h at room temperature. Chromogen 3,3′‐diaminobenzidine tetrahydrochloride (Dojindo Laboratories, Kumamoto, Japan) was applied as a 0.02% solution containing 0.005% H_2_O_2_ in 50 mM ammonium acetate-citrate acid buffer (pH 6.0). Mayer’s hematoxylin was lightly counterstained and mounted on each section. Negative controls consisted of PBS containing 0.1% bovine serum albumin instead of the primary antibody. The degree of cytoplasmic staining was evaluated in three levels. The cells with no staining at all were defined as grade 0, cells with clear staining as grade +2, and cells with weak staining (between grades 0 and +2) as grade +1. Grades 0 and +1 were defined as low expression and grade +2 was defined as high expression. Intratumoral CD8-positive cytotoxic lymphocytes (CD8^+^) were counted using light microscopy at 200× magnification in three selected hotspots. We defined patients with a cytotoxic lymphocyte count greater than 10 as belonging to the high cytotoxic lymphocyte infiltration group in our previous study^31^. The Ki-67 labelling index was calculated as the percentage of positive tumor cell nuclei, using more than 500 tumor cells, regardless of staining intensity^31^.

### Cell cultures

In this study, we used the human hepatoblastoma cell line HepG2 and the human HCC cell lines Hep3B, PLC/PRF/5 (PLC), Huh7, HLE, and HLF. Cell lines were purchased from the JCRB Cell Bank and American Type Culture Collection (Manassas, VA, USA). All cells used in the experiments were free of mycoplasma contamination. The cells were cultured in Dulbecco’s modified Eagle’s medium (Wako, Richmond, VA, USA) supplemented with 1% penicillin/streptomycin and 10% fetal bovine serum. All cells were cultured in humidified incubators with 5% CO_2_ at 37 °C.

### Knockdown of RNF31

RNF31-specific siRNAs (Supplementary Table S3) and negative control siRNA were purchased from Dharmacon GE Healthcare (Lafayette, CO, USA). HepG2, Hep3B, and PLC/PRF/5 cells were suspended at a density of 5.0 × 10^5^ cells in 50 μL Opti-MEM I reduced serum media (Thermo Fisher Scientific, Waltham, MA, USA) and then mixed with RNF31-specific siRNA or negative control siRNA. Transfection with siRNA was performed using a CUY21 EDIT II electroporator (BEX Co. Ltd., Tokyo, Japan), with poring and transfer pulses applied at 125 V and 10 V, respectively.

### Luciferase assay

The pGL4-NF-κB-Luc plasmid was co-transfected with the pGL4-Renilla-Luc/TK plasmid. The activity of luciferase was measured 24 h after transfection by lysing the cells using a GloMax 20/20 luminometer (Promega, Madison, WI, USA) and the dual-luciferase reporter assay system (Promega). Cells were stimulated with TNF-α (Promega) at 10 ng/mL for 6 h.

### Cell proliferation assay

Cells were cultured in 96-well culture plates at a density of 2000 cells/well in 100 μL of medium. Cell viability was analyzed using CCK-8 (Dojindo Laboratories, Kumamoto, Japan). Evaluations were performed at 24, 48, 72, and 96 h. The cell counting solution was added at a concentration of 10 μL/well, and cells were incubated at 37 °C in a humidified 5% CO_2_ atmosphere for 2 h and 30 min. The optical density of the wells was measured at 450 nm using a spectrophotometer (Bio-Rad Laboratories, Hercules, CA, USA). HOIPIN-8 (Axon2972) was purchased from Axon MedChem (Reston, VA, USA).

### Cell invasion assay

Cell invasion was evaluated using 24-well Corning BioCoat Matrigel invasion chambers (Corning, Corning, NY, USA). The lower chamber was filled with medium containing 10% or 20% fetal bovine serum, and the upper chamber was seeded with cells in serum-free Dulbecco’s modified Eagle’s medium. After 48 h, the cells were fixed and stained with Diff-Quik (Sysmex, Hyogo, Japan). Cell invasion was assessed by counting the number of cells in five random fields observed under a microscope.

### Immunoblotting analysis

Protein extraction was performed using a lysis buffer containing 50 mM Tris–HCl (pH 7.5), 150 mM NaCl, 1% Triton X-100, and a complete protease and phosphatase inhibitor cocktail for 20 min on ice. SDS-PAGE with 10% Bis–Tris gels was used to separate the proteins, which were then transferred to nitrocellulose sandwiches (#12369; Cell Signaling Technology, Danvers, MA, USA). After blocking with 5% bovine serum albumin or 5% skim milk for 1 h at room temperature, the membranes were incubated with primary antibodies overnight at 4 °C. A membrane used for detection of multiple proteins were cut prior to incubation with primary antibodies (i.e. a membrane would be cut at the height of 75 kilodalton, then the upper membrane used for immunoblotting of RNF31 and the lower membrane for RBCK1). The following antibodies were used for immunoblotting (all obtained from Cell Signaling Technology): phospho-IκBα (1:1000; 9246); IκBα (1:1000; 4812); phospho-p105 (1:1000; 4806); p105 (1:1000; 13586); phospho-p65 (1:1000; 3033); p65 (1:1000; 8242); PARP (1:1000; 9542); Caspase-3 (1:1000; 9665); and cleaved-caspase-3 (1:1,000; 9664). Additionally, β-actin (1:5000; A5316; Sigma-Aldrich, St. Louis, MO, USA), HOIP (1:1,000; SAB2102031; Sigma-Aldrich); RBCK1 (1:1000; NBP1-88301; Novus Biologicals, Englewood, CO, USA); SHARPIN (1:1000; 14626-1-AP; Proteintech, Rosemont, IL, USA); and α-tubulin (1:5000; CLT9002; Cedarlane) antibodies were used for immunoblotting. The membranes were treated with horseradish peroxidase-linked secondary antibodies and imaged using an ImageQuant LAS 4000 instrument (GE Healthcare, Chicago, IL, USA) and ECL Prime Western blotting detection system (GE Healthcare).

### Reverse-transcription quantitative polymerase chain reaction

RNA was extracted using an RNeasy Mini Kit (#217004; Qiagen, Venlo, the Netherlands) and quantified using an ND-1000 spectrophotometer (NanoDrop Technologies, Wilmington, DE, USA). Reverse-transcription (RT) quantitative polymerase chain reactions (qPCRs) were performed using an RT kit (Toyobo, Osaka, Japan) and Power SYBR Green PCR master mix (Life Technologies, Rockville, MD, USA) according to the manufacturer’s instructions. The qPCR was performed with a Step-One-Plus PCR system (Applied Biosystems, Wilmington, DE, USA) using the 2^−ΔΔCt^ method. The primers used are listed in Supplementary Table S4. GAPDH was used to normalize RNA input for all RT-qPCR analyses.

### Statistical analysis

Statistical significance was analyzed using the Mann–Whitney U test, analysis of variance, or Welch’s t-test for continuous variables; the χ^2^ test or Fisher’s exact test was performed for categorical variables. When the analyses of variance results were significant, Tukey’s multiple comparison tests were performed to assess the differences between groups. Survival curves were calculated using the Kaplan–Meier method. Differences between the survival curves were analyzed using log-rank tests. Prognostic factors were examined using univariate and multivariate analyses and the Cox proportional hazards model. Results were considered statistically significant when the relevant *P* value was < 0.05, and all statistical analyses were performed using the JMP 15 software (SAS Institute, Cary, NC, USA).

### Ethics approval and consent to participate

This study was conducted in compliance with the principles of the Declaration of Helsinki. All patients were eligible for our study in accordance with the institutional guidelines of Gunma University Hospital (approval number: HS2020-124). Patient consent was obtained by using the opt-out method.

### Supplementary Information

Supplementary Information 1.

Supplementary Information 2.

Supplementary Information 3.

Supplementary Information 4.

Supplementary Information 5.

Supplementary Information 6.

Supplementary Information 7.

Supplementary Information 8.

Supplementary Information 9.

Supplementary Information 10.

## Data Availability

Data supporting the findings of this study are available from the corresponding author upon request.
