# Supplementary figures and images for: RNF31 promotes proliferation and invasion of hepatocellular carcinoma via nuclear factor kappaB activation
Source: Sci Rep. 2024 Jan 3;14:346. doi: 10.1038/s41598-023-50594-3 (PMC10764851; doi:10.1038/s41598-023-50594-3)

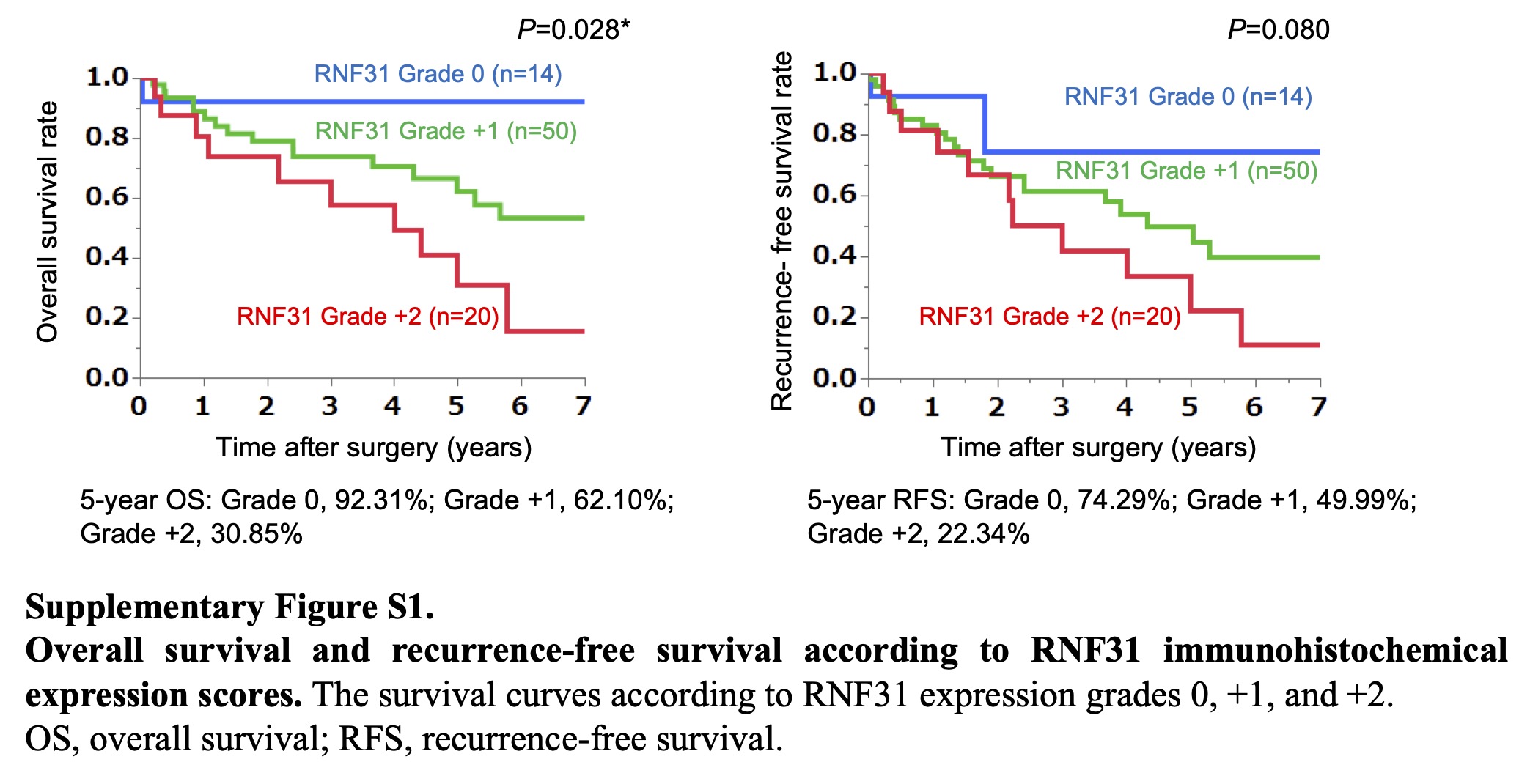

Supplement: Supplementary file 1 — Supplementary Information 1. [file 41598_2023_50594_MOESM1_ESM.jpg]

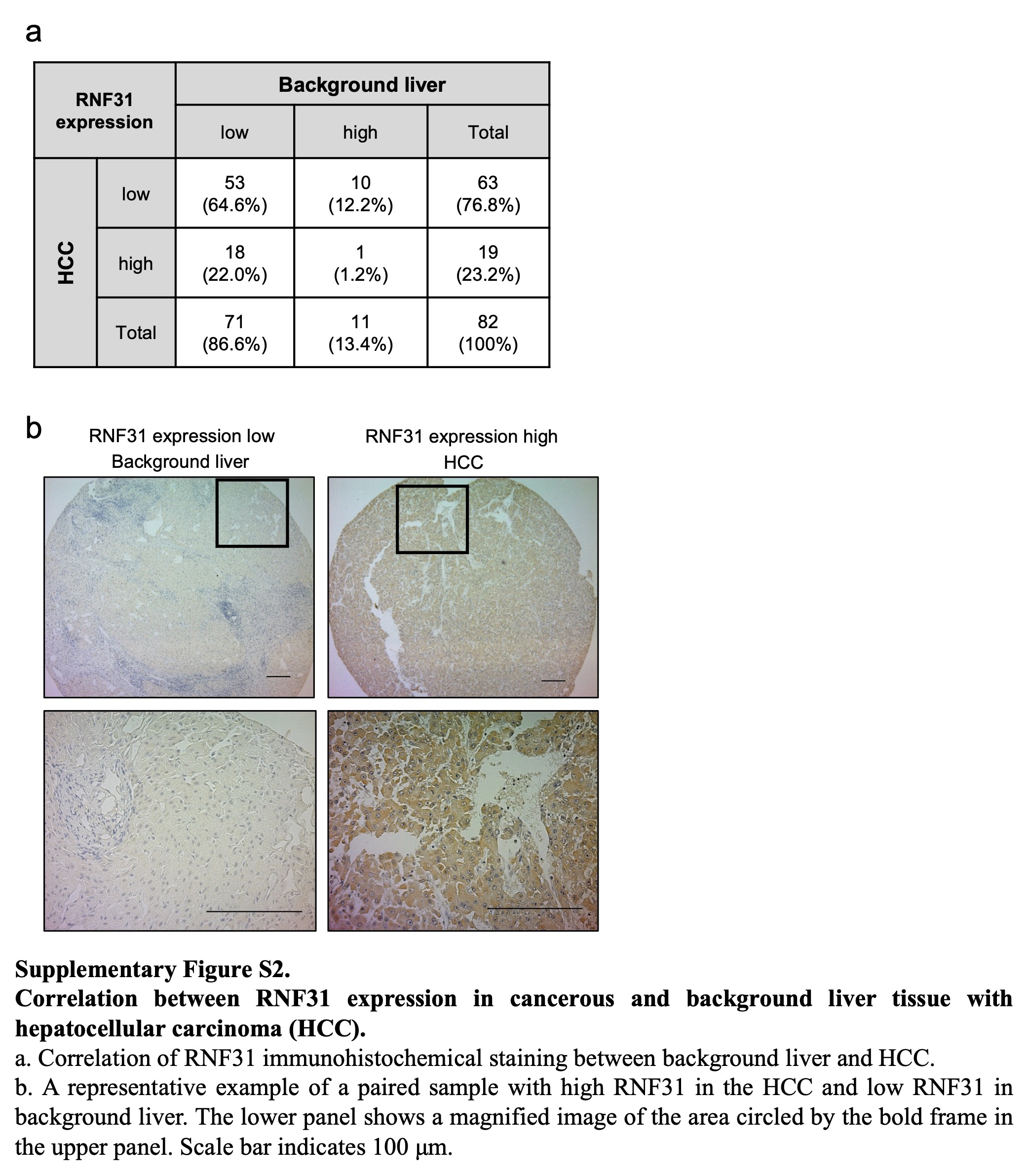

Supplement: Supplementary file 2 — Supplementary Information 2. [file 41598_2023_50594_MOESM2_ESM.jpg]

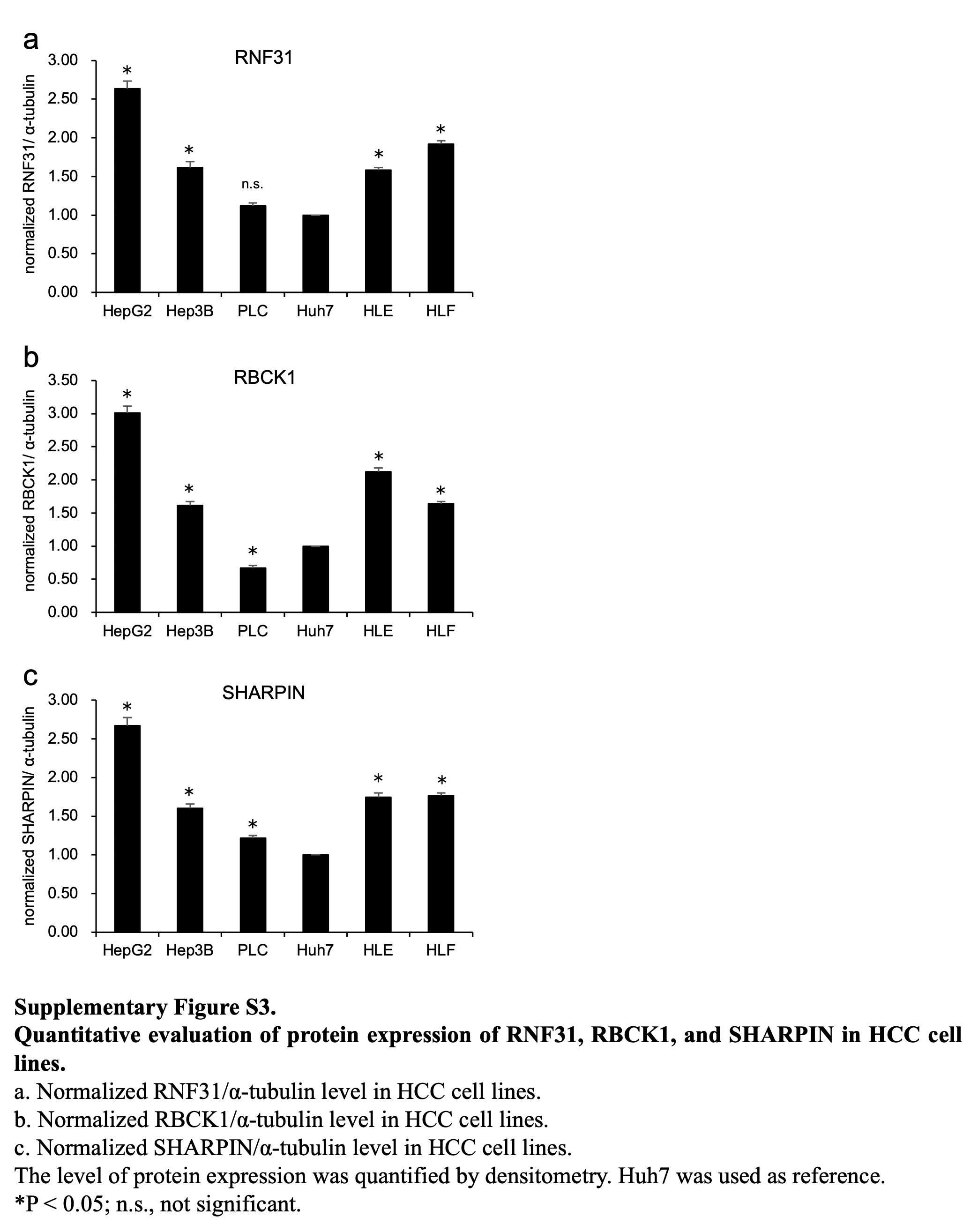

Supplement: Supplementary file 3 — Supplementary Information 3. [file 41598_2023_50594_MOESM3_ESM.jpg]

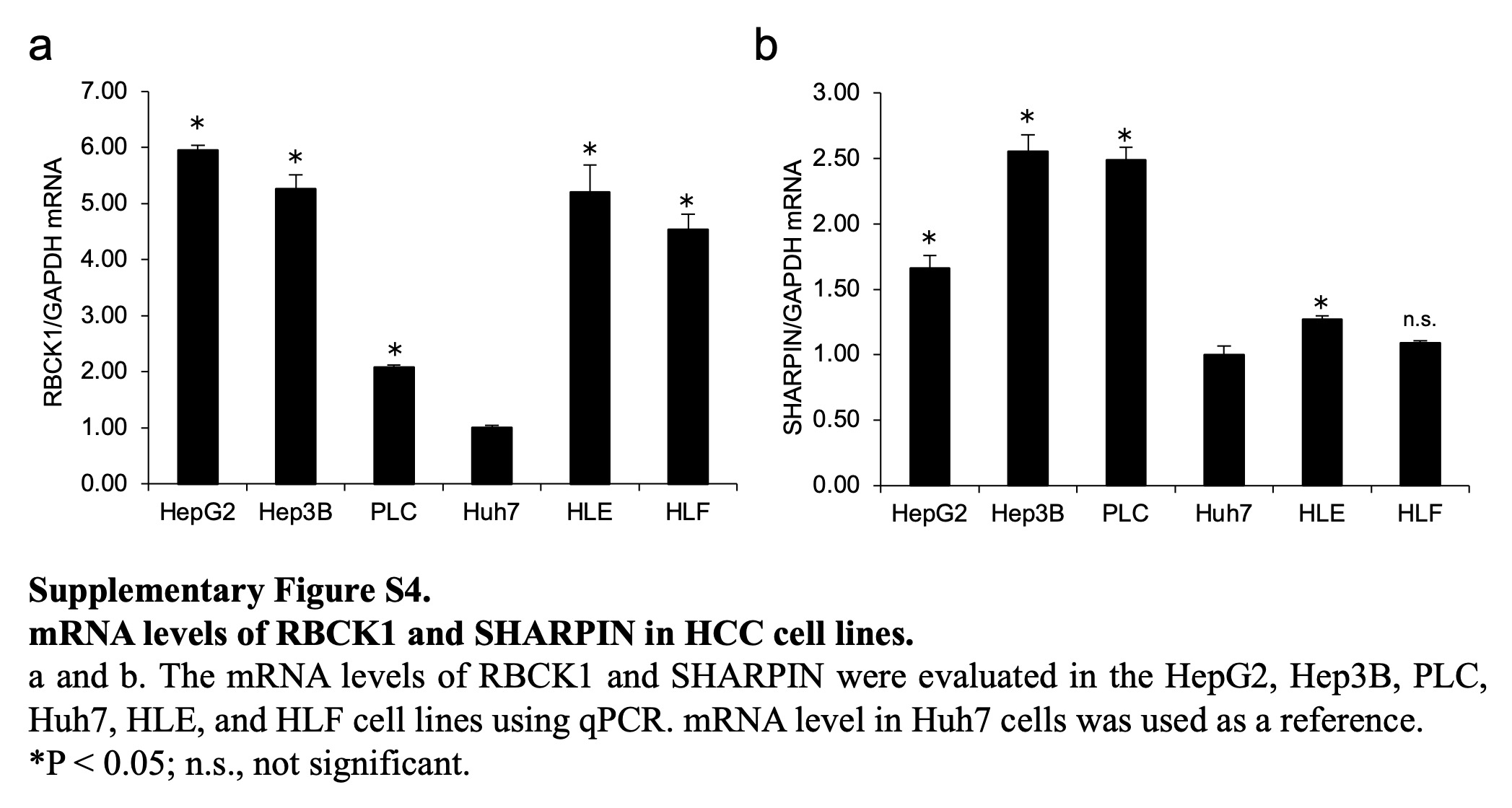

Supplement: Supplementary file 4 — Supplementary Information 4. [file 41598_2023_50594_MOESM4_ESM.jpg]

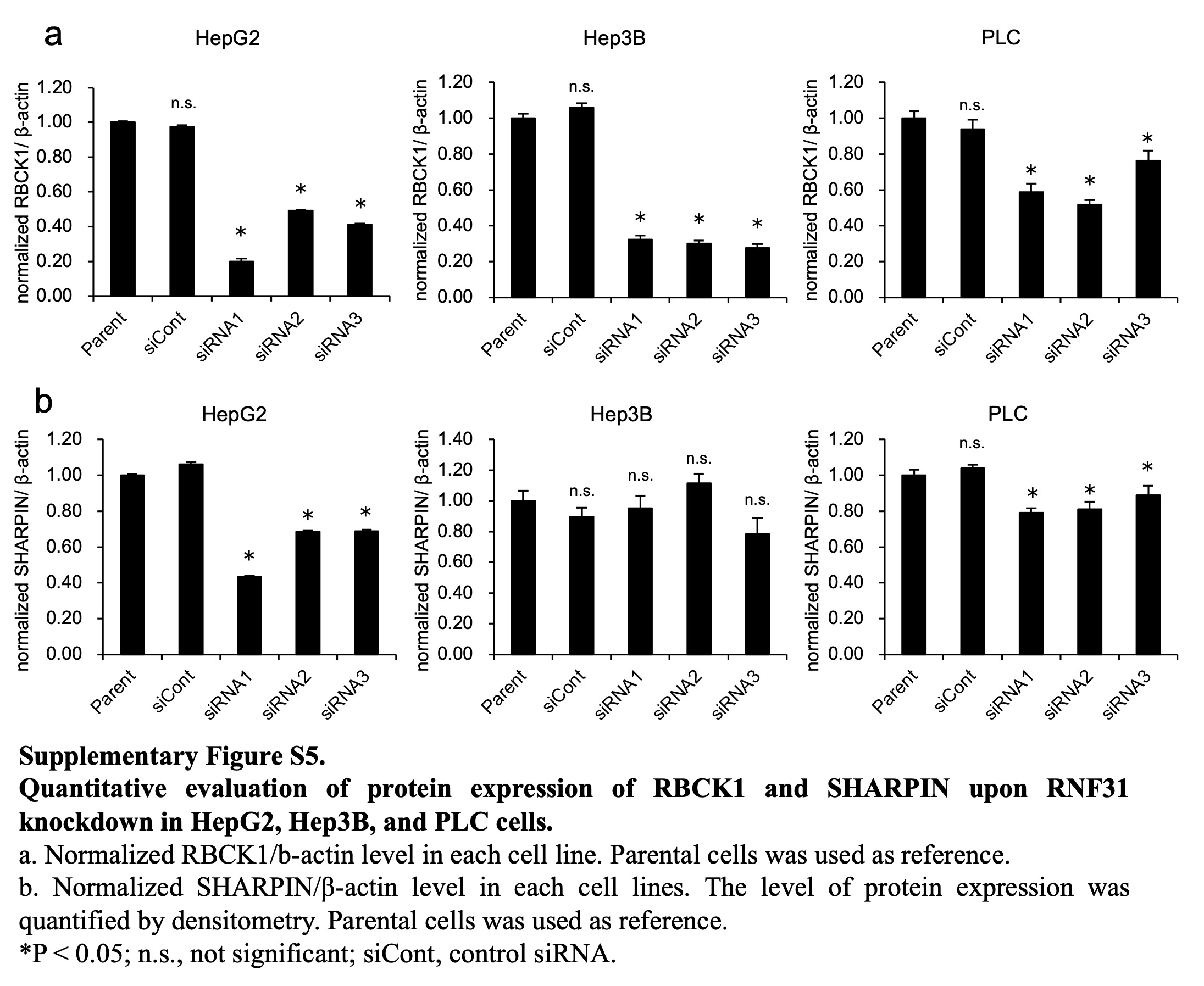

Supplement: Supplementary file 5 — Supplementary Information 5. [file 41598_2023_50594_MOESM5_ESM.jpg]

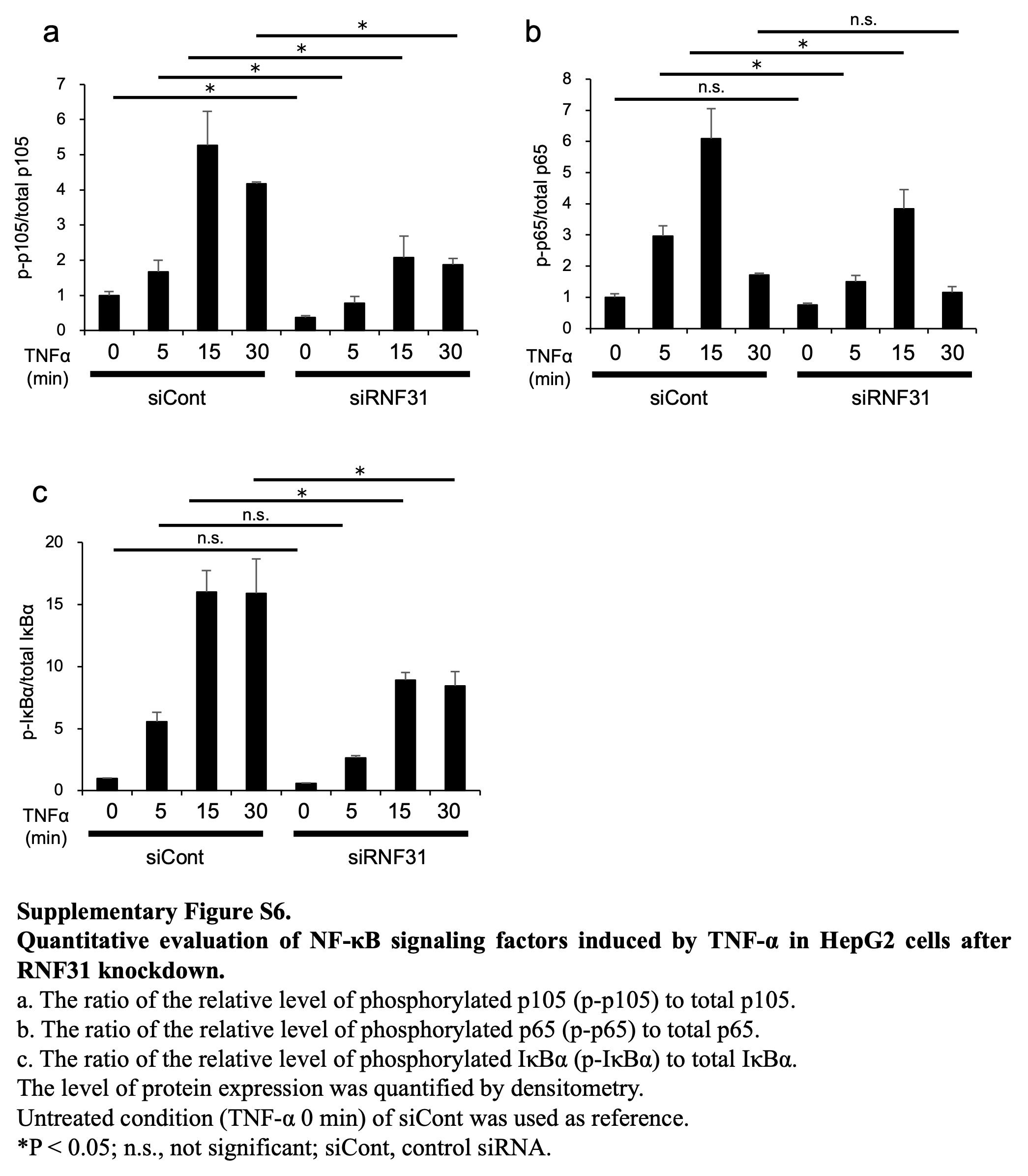

Supplement: Supplementary file 6 — Supplementary Information 6. [file 41598_2023_50594_MOESM6_ESM.jpg]

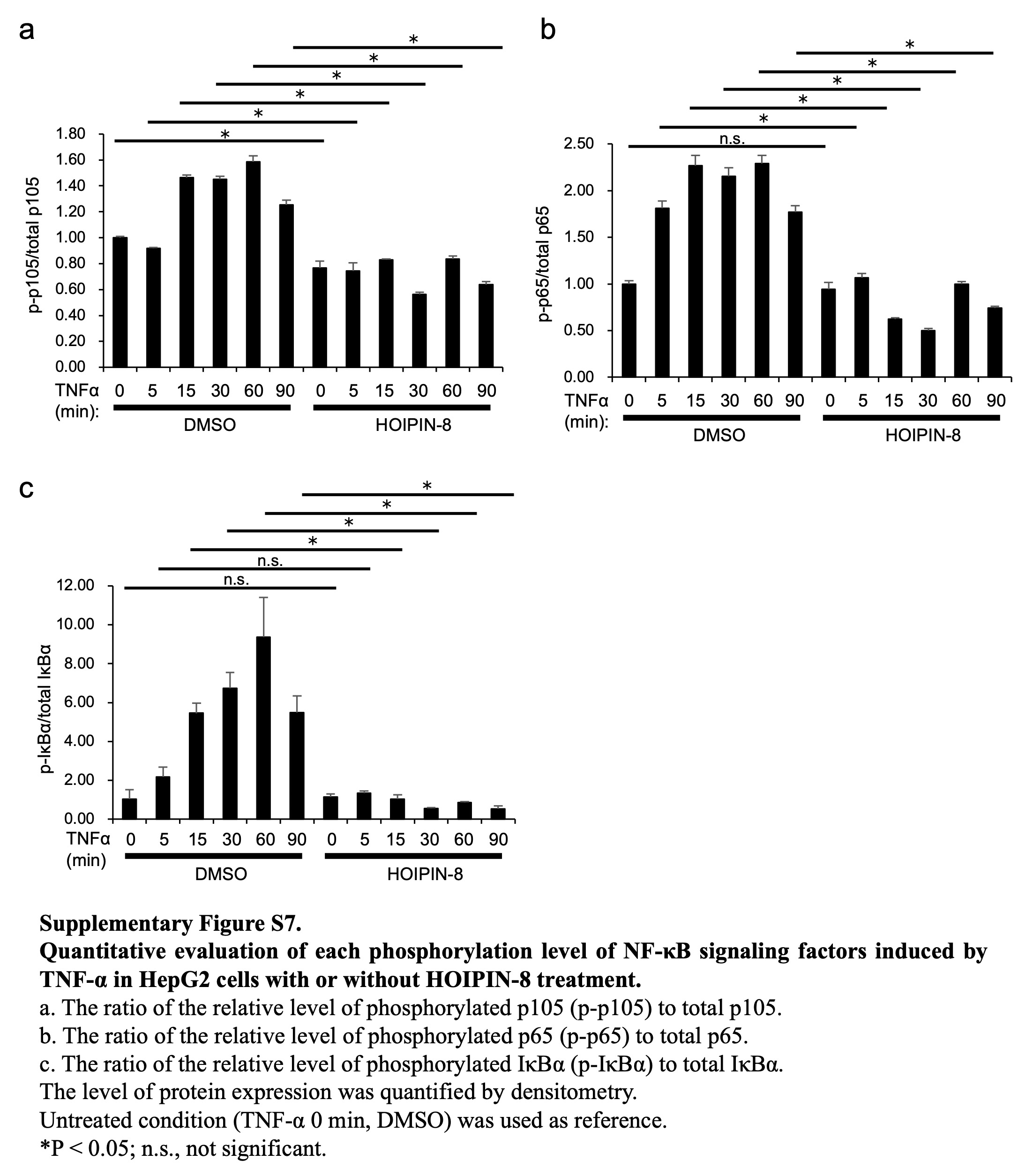

Supplement: Supplementary file 7 — Supplementary Information 7. [file 41598_2023_50594_MOESM7_ESM.jpg]

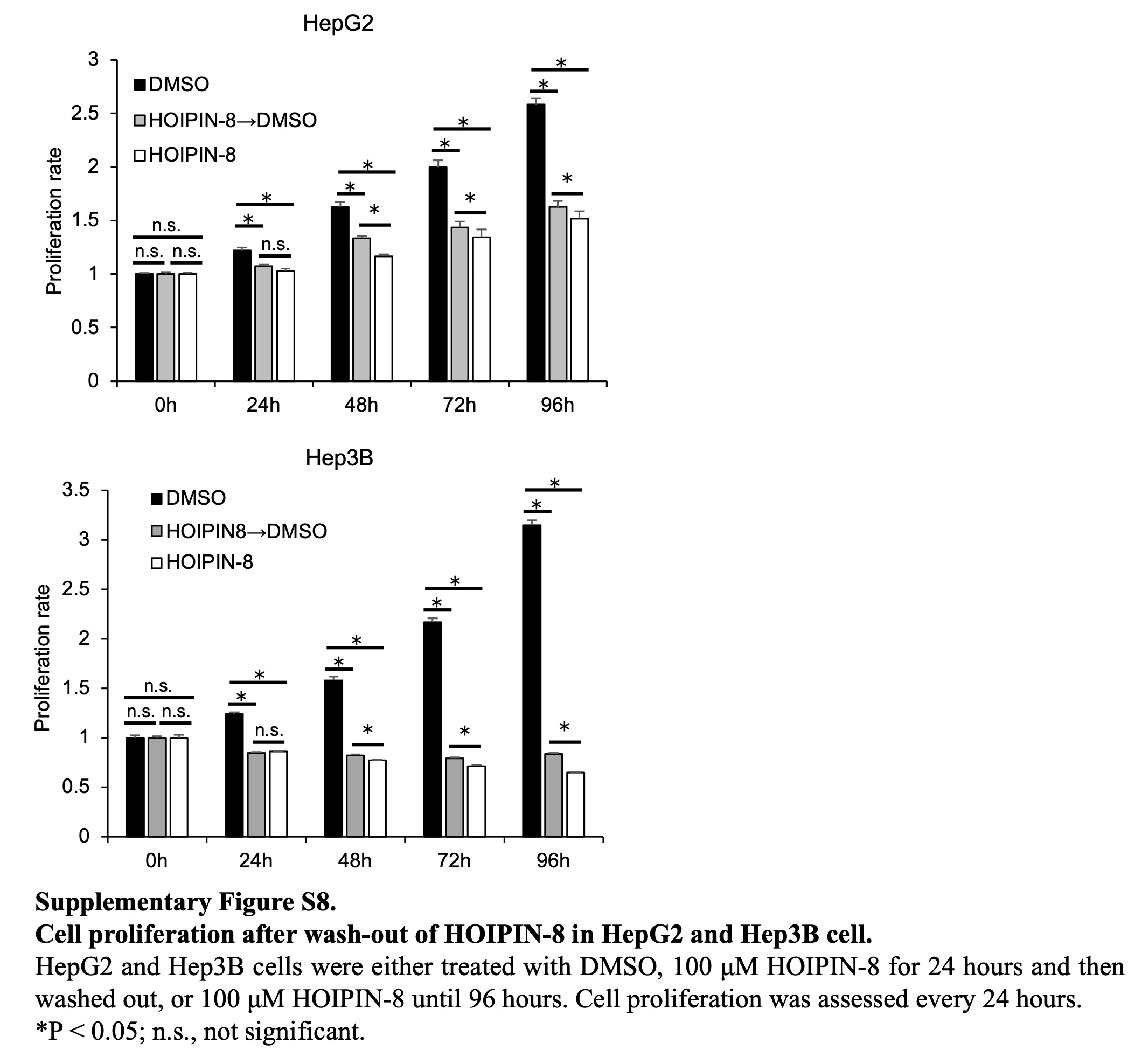

Supplement: Supplementary file 8 — Supplementary Information 8. [file 41598_2023_50594_MOESM8_ESM.jpg]

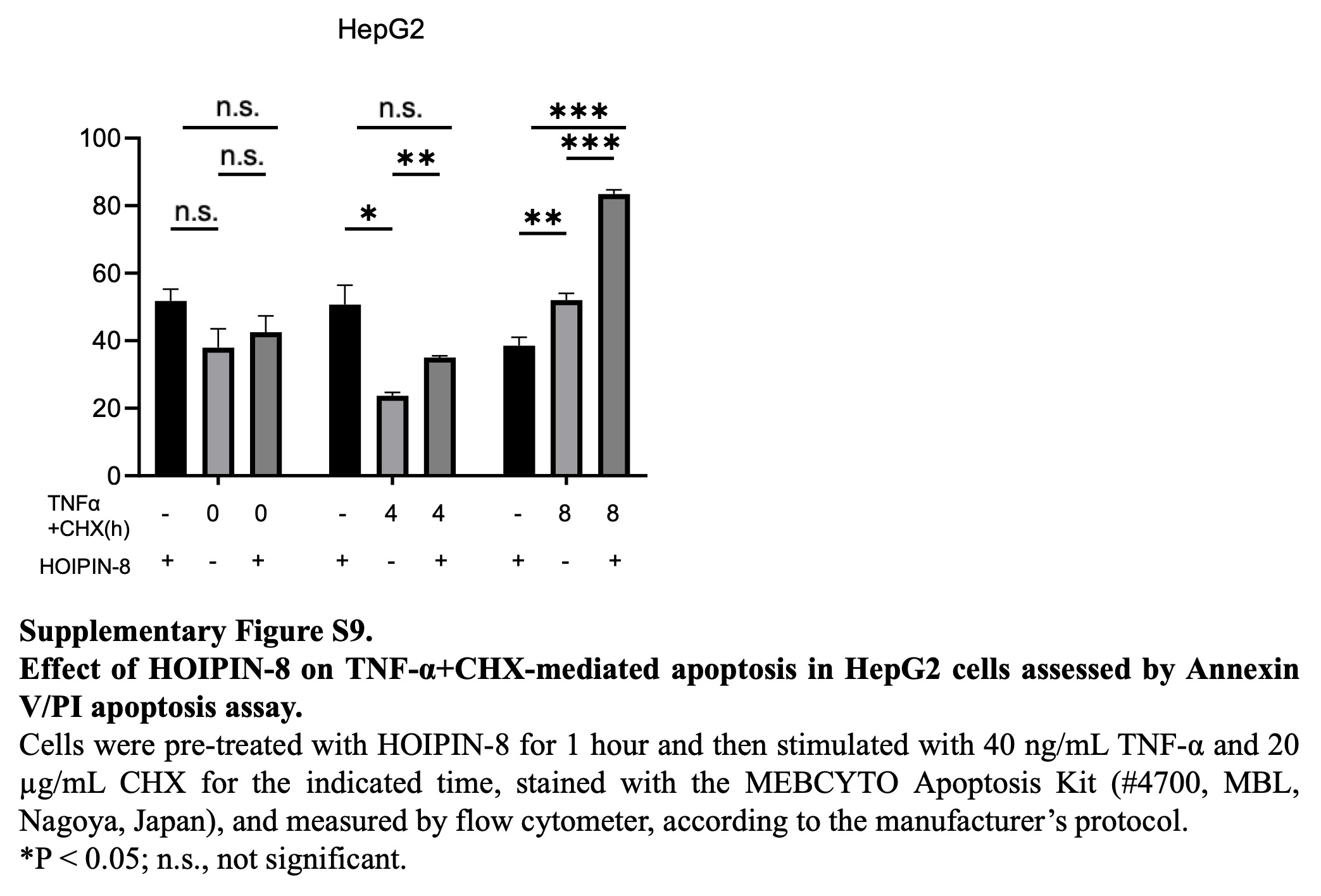

Supplement: Supplementary file 9 — Supplementary Information 9. [file 41598_2023_50594_MOESM9_ESM.jpg]
