# Supplementary material for: RNF31 promotes proliferation and invasion of hepatocellular carcinoma via nuclear factor kappaB activation
Source: Sci Rep. 2024 Jan 3;14:346. doi: 10.1038/s41598-023-50594-3 (PMC10764851; doi:10.1038/s41598-023-50594-3)
Supplement: Supplementary file 10 — Supplementary Information 10. [file 41598_2023_50594_MOESM10_ESM.docx]

| Supplementary Table S1. Clinicopathological characteristics according to RNF31 expression in background liver tissue. | | | |
| --- | --- | --- | --- |
| Patient characteristics | RNF31 expression in background liver tissue | | *P* value |
|  | Low expression  (n = 71) | High expression  (n = 11) |  |
| Age (y) |  |  |  |
| ≤65 | 20 | 3 | 0.951 |
| >65 | 51 | 8 |  |
| Sex |  |  | 0.729 |
| Male | 55 | 8 |  |
| Female | 16 | 3 |  |
| Viral hepatitis |  |  | 0.588 |
| Negative | 32 | 4 |  |
| Positive | 39 | 7 |  |
| Liver cirrhosis |  |  |  |
| Negative | 45 | 6 | 0.574 |
| Positive | 26 | 5 |  |
| Differentiation |  |  | 0.656 |
| Well or moderate | 67 | 10 |  |
| Poor | 4 | 1 |  |
| T classification |  |  | 0.182 |
| 1 | 7 | 0 |  |
| 2 | 24 | 1 |  |
| 3 | 32 | 8 |  |
| 4 | 8 | 2 |  |
| Tumor growth pattern |  |  | 0.030* |
| Expansive growth | 59 | 6 |  |
| Invasive growth | 12 | 5 |  |
| Cancerous capsule infiltration |  |  | 0.842 |
| Negative | 41 | 6 |  |
| Positive | 30 | 5 |  |
| Intrahepatic metastasis |  |  | 0.917 |
| Negative | 59 | 9 |  |
| Positive | 12 | 2 |  |
| Portal vein invasion |  |  | 0.005* |
| Negative | 50 | 3 |  |
| Positive | 21 | 8 |  |
| Hepatic vein invasion |  |  | 0.396 |
| Negative | 57 | 10 |  |
| Positive | 14 | 1 |  |
| Hepatic artery invasion |  |  | 0.028* |
| Negative | 69 | 9 |  |
| Positive | 2 | 2 |  |
| Tumor size (mm) |  |  | 0.241 |
| ≤20 | 8 | 0 |  |
| >20 | 63 | 11 |  |
| AFP (ng/mL) | 933± 4803 | 82 ± 185 | 0.154 |
| PIVKA-II (mAU/mL) | 8704 ± 42909 | 3419 ± 7234 | 0.375 |
| CD8 infiltration |  |  | 0.578 |
| Low (≤10) | 20 | 4 |  |
| High (>10) | 51 | 7 |  |
| Ki-67 labelling index |  |  | 0.509 |
| ≤10 | 59 | 10 |  |
| >10 | 12 | 1 |  |
| AFP, α-fetoprotein; PIVKA-II, protein induced by vitamin K absence II. **P* < 0.05 | | | |

| Supplementary Table S2. IC50 values of HOIPIN-8 in HepG2 and Hep3B cell lines. | | | | |
| --- | --- | --- | --- | --- |
| IC50 (µM) | 24 hrs | 48 hrs | 72 hrs | 96 hrs |
| HepG2 | 91.6 | 86.6 | 85.1 | 94.4 |
| Hep3B | 100.8 | 86.7 | 79.8 | 72.5 |
| IC50, 50% inhibitory concentration. | | | | |

| Supplementary Table S3. List of siRNAs used for RNF31 knockdown. | |
| --- | --- |
| siRNA 1 | ACACACAGUUGCUCAGCUATT, UAGCUGAGCAACUGUGUGUTT |
| siRNA 2 | GGACGAAGCUUGUGGCAAGTT, CUUGCCACAAGCUUCGUCCTT |
| siRNA 3 | GCUCCAGCACCAAUAAAGATT, UCUUUAUUGGUGCUGGAGCTT |

| Supplementary Table S4. List of primers used for quantitative polymerase chain reactions. | | |
| --- | --- | --- |
|  | Forward primer | Reverse primer |
| *RNF31* | 5’-CTTCTGTGTGCGCTGCAA-3’ | 5’-TTCTGGAAGTCCTCACAGCTC-3’ |
| *RBCK1* | 5’-AGTGTCTGCACACCTTCTGC-3’ | 5’-GCACGAGTAGGTGTTGTCAATG-3’ |
| *SHARPIN* | 5’-CCCTGCTTTCCTCTACTTGC-3’ | 5’-GCTGAGGGCTAGGTCCTGT-3’ |
| *IL-6* | 5’- AGCCACTCACCTCTTC-3’ | 5’GCCTCTTTGCTGCTTT-3’ |
| *IL-8* | 5’-TTTTGCCAAGGAGTGCTAAAGA-3’ | 5’-AACCCTCTGCACCCAGTTTTC-3’ |
| *BIRC3* | 5’-AGATGAAAATGCAGAGTCATCAAT-3’ | 5’-CATGATTGCATCTTCTGAATGG-3’ |
| *GAPDH* | 5’-AACCCTCTGCACCCAGTTTTC-3’ | 5’-GTGTGGTGGGGGACTGAG-3’ |
